# Electrochemical Microwell Plate to Study Electroactive Microorganisms in Parallel and Real-Time

**DOI:** 10.3389/fbioe.2021.821734

**Published:** 2022-02-15

**Authors:** Anne Kuchenbuch, Ronny Frank, José Vazquez Ramos, Heinz-Georg Jahnke, Falk Harnisch

**Affiliations:** ^1^ Department of Environmental Microbiology, UFZ—Helmholtz-Centre for Environmental Research GmbH, Leipzig, Germany; ^2^ Centre for Biotechnology and Biomedicine, Molecular Biological-Biochemical Processing Technology, Leipzig University, Leipzig, Germany

**Keywords:** microbial electrochemical technology, electroactive microorganisms, microbial ecology, multipotentiostat, microbial resource mining

## Abstract

Microbial resource mining of electroactive microorganism (EAM) is currently methodically hampered due to unavailable electrochemical screening tools. Here, we introduce an electrochemical microwell plate (ec-MP) composed of a 96 electrochemical deepwell plate and a recently developed 96-channel multipotentiostat. Using the ec-MP we investigated the electrochemical and metabolic properties of the EAM models *Shewanella oneidensis* and *Geobacter sulfurreducens* with acetate and lactate as electron donor combined with an individual genetic analysis of each well. Electrochemical cultivation of pure cultures achieved maximum current densities (*j*_max_) and coulombic efficiencies (*CE*) that were well in line with literature data. The co-cultivation of *S. oneidensis* and *G. sulfurreducens* led to an increased current density of *j*_max_ of 88.57 ± 14.04 µA cm^−2^ (lactate) and *j*_max_ of 99.36 ± 19.12 µA cm^−2^ (lactate and acetate). Further, a decreased time period of reaching *j*_max_ and biphasic current production was revealed and the microbial electrochemical performance could be linked to the shift in the relative abundance.

## Introduction

Microbial electrochemical technologies (MET) are an upcoming platform allowing the coupling of microbial and electrochemical conversions (Schröder et al., 2015). Thus, MET are considered an essential piece for establishing electrobiorefineries or Power-to-X in a future biobased, circular, and electrified economy (Harnisch and Urban 2018).

The foundation of primary MET are electroactive microorganisms (EAM) (Logan 2009). The metabolism of EAM is linked to Faradaic current flow at electrodes (Schröder et al., 2015) via extracellular electron transfer (EET). Thus the microbial electrochemical conversion of microbial metabolites that are the starting materials or substrates from a technical point of view can be achieved. These conversions are redox reactions and include reductions at the cathode as well as oxidations at the anode. As primary MET facilitate reactions at electrodes that cannot be achieved without EAM, these can be denominated as microbial electrocatalysts.

The most prominent EAM are *Shewanella oneidensis* and *Geobacter sulfurreducens*. *S. oneidensis* grows primarily as suspended cells or thin biofilms and releases flavin molecules (Marsili et al., 2008) that act as soluble redox shuttles for a mediated extracellular electron transfer (MEET) to carry electrons from the cell surface to an external electron acceptor like an anode. MEET permits *S. oneidensis* to oxidize lactate to acetate under anaerobic conditions while growing in planktonic state without being attached to the anode (Lanthier et al., 2008). Additionally to MEET *S. oneidensis* also performs direct extracellular electron transfer (DEET) via membrane bound cytochromes over even micrometer distances (Chong et al., 2021). Therefore, it produces conductive appendages being extensions of the outer cell membrane (Subramanian et al., 2018). Thus, if no mediator is present EET is only possible, if the suspended cells have at least temporary physical contact with the anode (Harrisa et al., 2010; Starwalt-Lee et al., 2021). This limitation and the insufficient mediator production are the main reasons that *S. oneidensis* cannot reach high current densities (Logan et al., 2019). *G. sulfurreducens* performs only DEET by transferring electrons via outer membrane proteins and highly conductive nanowires/pili to an external electron acceptor (Shi et al., 2016; Yalcin and Malvankar 2020) and achieves high current densities at different anode materials. Also DEET to other microbial species can be carried out, which is also called DIET (direct interspecies electron transfer), representing another layer of trophic interaction in complex microbial communities (Summers et al., 2010; Lovley 2017). In MET *Geobacter* cells are directly attached to the anode forming multilayer biofilms, while their electrochemical performance is increasing with biofilm thickness (Reguera et al., 2006) until biofilm maturation is reached. In particular, the conductive nanowires/pili enable *G. sulfurreducens* to transfer electrons efficiently (Logan et al., 2019), also over long distances of more than 50 µm (Semenec and Franks 2015; Clarke and Edwards 2020). Unlike *S. oneidensis*, *G. sulfurreducens* is able to oxidize acetate completely to CO_2_ under anaerobic conditions while performing EET (Caccavo et al., 1994; Bond and Lovley 2003). Thereby it is of note that studying these model EAM is currently not routinely possible using electrochemical microwell plates or similar devices allowing high-throughput screening.

In principle almost every redox reaction could be achieved using EAM if the microorganisms possess the needed metabolic inventory (Koch and Harnisch 2016a). The phylogenetic as well as metabolic diversity of EAM seems unlimited. In contrast to its great promise, the number of EAM that is known or can be even tapped is very limited. Apart from the need of a more precise definition of EAM (Koch and Harnisch 2016b) the main limitation is the following. Microbial resource mining of EAM using classical or well-established methods in microbiology is not possible. For selection of EAM, a strong and specific selection force must be provided by an electrode either acting as terminal electron acceptor (TEA), i.e., an anode or electron donor (ED), i.e., a cathode. Approaches that are based on screening for EAM using electrochromism of tungsten oxide (WO_3_) (Yuan et al., 2013), electrochemiluminescence (You et al., 2019), colorimetric (Wen et al., 2014; Zhou et al., 2015), and dielectrophoretic methods (Wang et al., 2019) are insufficient, as these are only surrogates for true microbial electrochemical activity at electrodes (Yee et al., 2020) as using minerals as TEA does not mean that necessarily electrodes can be used as well (Rotaru et al., 2015).

**FIGURE 1:**
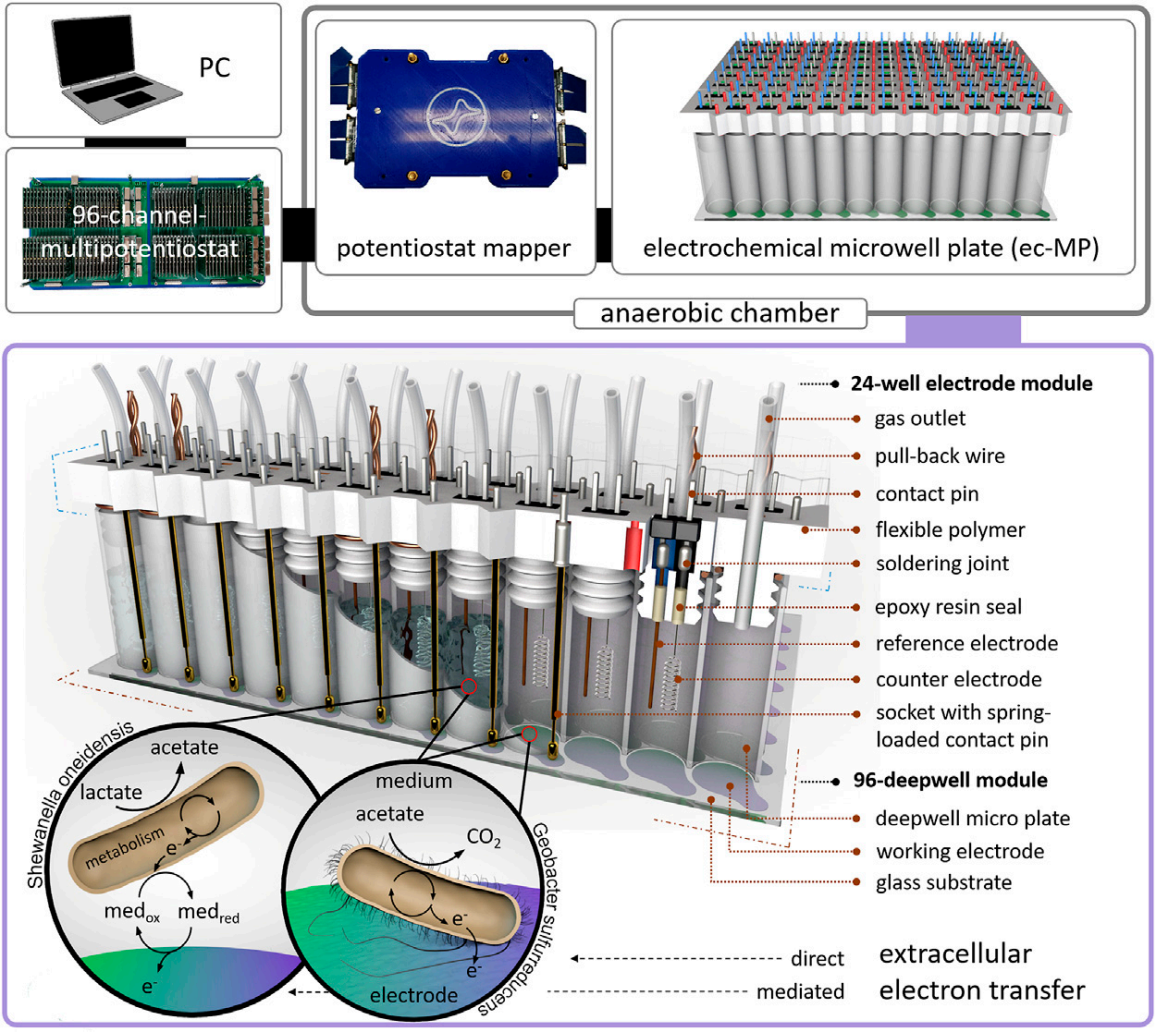
Design of the electrochemical microwell plate (ec-MP): The ec-MP consists of a 96-deepwell module and up to four 24-well electrode modules connected to a 96-channel multipotentiostat by a potentiostat mapper. In this study microbial electrochemical activity of *S. oneidensis* and *G. sulfurreducens* is measured as an example. Photographic images of single components can be found in Supplementary Figure S1.

Here, we present a 96-deepwell electrochemical microplate coupled with a recently developed 96-channel multipotentiostat (Frank et al., 2020) as a screening platform for EAM that is further denominated as an electrochemical microwell plate (ec-MP). Compared to our previous work, which only addressed enzymatic electrocatalysis, the design of the ec-MP used here enables long-term microbial electrochemical measurements under anaerobic conditions in small but sufficiently large volumes for biofilm formation. The ec-MP allows the parallelized and fully independent investigation of up to 96 electrochemical cells in ANSI standard well format with each well hosting one independent three-electrode arrangement being measured without multiplexing or the use of a capacitor circuit (Li et al., 2017). The ec-MP is based on an adapted 96-deepwell plate providing 96 electrochemical reaction chambers of 1.0–1.2 mL volume, here hosting indium tin oxide (ITO) working electrodes of 50 mm^2^, and allowing chronoamperometric and cyclic voltammetric as well as open cell potential (OCP) measurements for up to weeks under oxygen-free conditions (Figure 1). Thus, it enables potentiostatic control in each well compared to purely passive voltage sensing in 2-electrode arrangements (Szydlowski et al., 2022). Noteworthy, this is also clearly different and advantageous when being compared to previous work (Kumashi et al., 2021; Molderez et al., 2021) that offers a high degree of parallelization in terms of working electrodes, but makes use of a shared reaction chamber. Thus, the ec-MP introduced here also allows the study of simultaneously and truly independently electroactive pure and mixed cultures over time, for example, in different media or at different pH. It further allows chemical as well as genetic analysis of each electrode chamber as presented below. Given that the ec-MP operates with true replicates, providing independent reference electrodes, it is less prone to systematic failures when being compared to systems with a single reference electrode for all working electrodes. Moreover, the ec-MP allows multi-parametric analysis in contrast to paper-based approaches (Tahernia et al., 2020a), which are typically just for short-time use.

## Materials and Methods

### Chemicals

All chemicals were of least analytical grade and gases with a purity of least 99.8% were used. For all experiments deionized water (Millipore) was used. The potentials are provided vs. standard hydrogen electrode (SHE) by conversion from open Ag/AgCl (+ 0.4 vs. SHE) (see section Assembly and operation of the electrochemical microwell plate).

### Microorganisms, Media, and Pre-cultivation

The strains *Geobacter sulfurreducens* PCA (ATCC 51573, DSM 12127) and *Shewanella oneidensis* MR-1 (ATCC 700550) were used in this study.

*G. sulfurreducens* PCA was cultivated in minimal medium DSM 826 containing 0.82 g L^−1^ Na-acetate, 8.0 g L^−1^ Na_2_-fumerate, 1.50 g L^−1^ NH_4_Cl; 0.60 g L^−1^ Na_2_HPO_4_; 0.10 g L^−1^ KCl; 2.5 g L^−1^ NaHCO_3_ and 10 mL L^−1^ trace- and vitamin solution 141 (Supplementary Table S1). To achieve anaerobic growth of the planktonic pre-cultures the medium was gassed with 80/20 (v/v) N_2_/CO_2_. The pre-cultures were cultivated in 100 mL serum bottles with an oxygen tight butyl rubber stopper at 30°C at 150 rpm with a pH 6.8 for 5–7 days.

*S. oneidensis* MR-1 was cultivated in minimal medium M4 containing 0.221 g L^−1^ K_2_HPO_4_; 0.099 g L^−1^ KH_2_PO_4_; 1.19 g L^−1^ HEPES; 8.766 g L^−1^ NaCl; 1.189 g L^−1^ (NH_4_)_2_SO_4_; 0.246 g L^−1^ MgSO_4_ x 7H_2_O; 8.44 ml L^−1^ Na-lactate (50%); 13.14 g L^−1^ Ferric (III) Citrate; 0.168 g L^−1^ NaHCO_3_; 10 mL L^−1^ M4 trace element chloridesolution and 1 mL L^−1^ M4 trace element sulfate solution (Supplementary Table S1). The aerobic pre-cultivation was performed in 100 mL Erlenmeyer flask at 30°C, 150 rpm, and a pH of 7–7.4 for 24 h.

For cultivation in ec-MP modified medium DSM 826 without fumarate was used for both *G. sulfurreducens* and *S. oneidensis*. As electron donor 10 mmol L^−1^ acetate; 10 mmol L^−1^ lactate or 5 mmol L^−1^ acetate and 5 mmol L^−1^ lactate were provided, as indicated (Supplementary Table S2). After pre-cultivation the microbial cultures were centrifuged at 8.000 rpm and 4°C for 10 min in 50 mL Falcon tubes. The cell pellets were resuspended in DSM 826 without fumarate with the respective electron donor to a defined optical density at 600 nm (*OD*_600_) of 0.1. In the case of electrochemical co-cultivation of *G. sulfurreducens* and *S. oneidensis* the cultures were mixed in a ratio of 1:1 (v/v). A volume of 1 mL was used for each well of the ec-MP. The volume was transferred with sterile disposables syringes from anaerobic and sealed culture bottles to the deepwell module.

### Setup of the Electrochemical Microwell Plate and Microbial Electrochemical Experiments

#### Fabrication of 96-Deepwell Module

Indium tin oxide (ITO) working electrodes were produced via lift-off technique in a cleanroom (class 1000) using borosilicate glass substrates (113.5 × 75 × 1 mm^3^, Goettgens Industriearmaturen; Germany). Glass substrates were cleaned in piranha solution and structured with positive photo resist AR-P 3510 (Allresist, Germany). ITO (Sindlhauser Materials GmbH, Germany) was sputtered to a thickness of 350 nm using a CREAMET 500 (Creavac, Germany) and annealed at 500°C for 10 min.

The 96 electrode array was bonded to the top of a modified polypropylene 96-deepwell microwell plate (2 ml Riplate®, Ritter Medical, Supplementary Figure S2) using epoxy resin EPO-TEK 302-3M (Epoxy Technologies, Germany). Just before use, ITO electrodes were cleaned in 1 N NaOH for 15 min and washed with ultrapure water.

#### Fabrication of the 24-Well Electrode Module

The corpus of the module (Supplementary Material) was designed in Inventor Professional 2016 and was 3D printed using a flexible polymer (TPU filament, Ultimaker) on an Ultimaker 3 extended (Ultimaker, Netherlands). Platinum counter electrodes (Chempur GmbH, Germany) and silver wires (Advent Research Materials, England) for the reference electrodes (open Ag/AgCl) were soldered to a double contact-pin, inserted in the corpus, and sealed on the lamella plug site of the corpus with epoxy resin (EPO-TEK 302-3M). Spring loaded contact pins of a length of 45 mm and a hub of 4 mm were soldered to single sockets and plugged into the corpus. Sockets and contact-pins were soldered to wires that are connected to the potentiostat mapper. For each well a PTFE tube (outer diameter = 1.59 mm, inner diameter = 0.75 mm, Techlab GmbH, Germany) was inserted into the corpus as a gas outlet and capped on the outside of the well to a 3D-printed sealing strip. The 24-well modules were manually assembled to the deepwell module. High mechanical resistance between deepwell plate and lamella plugs yielded the contact pressure for the spring-loaded contact pin. Disassembly of the modules was possible by six pull-back wires each wrapped around two lamella plugs. The potentiostat mapper was connected to the 96-channel multipotentiostat via LSHM plugs (Samtec, United States). Details concerning the 96-channel multipotentiostat are described elsewhere (Frank et al., 2020).

#### Assembly and Operation of the Electrochemical Microwell Plate

To assemble the ec-MP the 96-deepwell module was placed in a fixation device and mounted with a mechanical load (Supplementary Material) in an anaerobic chamber (Supplementary Figure S3). Oxygen content was measured using the SevenExcellence DO meter S479-K (Mettler Toledo, United States) and was reduced by a continuous flow of N_2_ gas, until an oxygen-free environment was achieved. After inoculation the 24-well electrode modules were consecutively pressed into the 96-deepwell plate. Due to the tight fit between the 96-deepwell wall and the lamella plugs of the 24-well electrode modules, no further fixation was necessary to achieve the contact pressure for the spring-loaded contact pins to assure electrical contact. The individual electrochemical cells were connected to the multipotentiostat using a potentiostat mapper, which routes the single electrodes from the contact wires to the LSHM plugs in the potentiostat (Supplementary Figure S1) by a PCB. Chronoamperometric measurements were performed at 0.32 V vs. open Ag/AgCl in a medium containing a chloride concentration of 29.5 mM. The used potential of 0.32 V vs. open Ag/AgCl was determined by potentiometric measurement using a commercial reference electrode Ag/AgCl (sat. KCl; SE11 Sensortechnik Meinsberg, Germany) over a time period of 40 days. A stable offset of 0.12 V in average was measured. The potential of 0.32 V vs. open Ag/AgCl is corresponding to 0.4 V vs. SHE. To assure anaerobic conditions N_2_ flushing and O_2_ monitoring was regularly performed during the whole experiment in the anaerobic chamber. The microbial electrochemical cultivations are summarized in Supplementary Table S2. The chronoamperometric measurements were performed in consecutive 8 h intervals to avoid data loss considering the high data density (48 channels, 5 Hz) and length of an experiment. This required the re-initialization of the potentiostat modules, associated with the short-term occurrence of typical high capacitive currents (i.e., spikes).

### Chemical and Microbial Analysis

#### Sample Preparation

After the experiments the anaerobic chamber was opened, the ec-MP was disassembled, and samples for microbial as well as chemical analysis were taken. The developed anodic biofilm was resuspended with the planktonic phase of the respective well and the whole content (ca. 1 ml) was transferred in a 1.5 mL reaction tube, so that combined samples including planktonic and biofilm cells were obtained. The samples were centrifuged at 10,000 × g for 5 min. The liquid phase was analysed via HPLC and the cell pellet was used for microbial analysis. Also samples of the pure cultures as well as the mixed culture inoculum were analysed.

#### Microbial Analysis

The microbial composition on DNA level was analysed with a standard TRFLP procedure using the primers UniBac27f (FAM labeled) and Univ1492r for amplifying the partial sequence of the 16S rRNA gene of bacteria (Lane 1991). The extraction of genomic DNA was performed with the NucleoSpin Tissue Kit (Macherey-Nagel, Germany). The PCR MasterMix contained 6.25 µL enzyme mix (MyTaq HS Red Mix, 2x, Bioline, Germany), 0.25 µL of each primer (5 µmol µL^−1^, supplied by MWG Biotech, Germany), 3.75 µL nuclease-free water, and 2 µL genomic DNA (about 10–20 ng). The PCR cycle parameters were as follows: 1 min at 95°C, 25 cycles of 15 s at 95°C, 15 s at 54°C, and 2 min at 72°C, followed by a 10 min extension step at 72°C (Koch et al., 2014). PCR products were purified (Sure Clean Plus, Bioline) and digested with restriction endonucleases *Hae*III and *Rsa*I (New England Biolabs, Germany).

A terminal restriction fragment length polymorphism (TRFLP) analysis was performed by using an ABI PRISM Genetic Analyzer 3130xl (Applied Biosystems™) and MapMarker® 1000 (BioVentures Inc., United States) as size standard.

In the TRFLP profiles (Supplementary Figure S4) of the inoculum samples (t_0_) also other TRFs were detected. Assuming strict sterile cultivation conditions for non-electrochemical pre cultivation, the presence of additional TRFs indicates a possible technical problem. In the TRFLP profiles of the samples after the electrochemical experiments also further TRFs occurred. Therefore, the discussion of the TRFLP profiles is limited to the TRF 30 bp representing *S. oneidensis* and TRF 214 bp representing *G. sulfurreducens* (TRFs were generated *via Hae*III endonuclease restriction).

#### Chemical Analysis

HPLC samples were analysed to record the ED consumption. Acetate and lactate were determined using HPLC (Shimadzu Scientific Instruments, Kyoto, Japan) equipped with a refractive index detector RID-10A and a HiPlex H column 300 × 7.7 mm (Agilent Technologies, Inc. CA, United States) with a pre-column SecurityGuard Cartridge Carbo-H 4 × 3.0 mm (Phenomenex, United States). The liquid phase of the HPLC was 0.1 N H_2_SO_4_. The samples were run for 30 min isocratically at a flow rate of 0.5 mL min^−1^ at 50°C. Peak calibration and identification was carried out with external standards (four point calibration for acetate and lactate from 0.1 g L^−1^ to 1 g L^−1^, *R* ^2^_(acetate)_ = 0.999, *R* ^2^_(lactate)_ = 0.997).

An increased acetate concentration after the experiment could be observed in the abiotic as well as in the OCP controls and showed that over the long experiment duration evaporation has occurred. In average the concentration of the carbon source increased to 13%, this factor was used to correct the substrate consumption and thus for calculation the Coulombic efficiencies (*CE*).

### Data Analysis and Calculations

The Coulombic efficiencies (*CE*) were calculated from the consumed ED and produced charge (Eq. 1).

$$CE=\frac{\int_{0}^{t}Idt}{\Delta C\times V\times z\times F\;}$$ (1)

I is current in A, *t* is time in s, ΔC is change of ED concentration lactate or/and acetate in mol L^−1^, V is volume of 0.001 L, z is number of electrons (4 for oxidation of lactate to acetate, 8 for acetate to CO_2_), and F is Faraday constant (96,485.3 C mol^−1^).

We considered as time of maximum activity *t*_max_ the period from inoculation until the maximum current density *j*_max_ was reached.

## Results and Discussion

To test and benchmark the ec-MP we performed pure single culture as well as co-cultivation experiments using the model EAM *S. oneidensis* and *G. sulfurreducens*. Cultivation was conducted at 0.4 V vs. standard hydrogen electrode (SHE). Controls at OCP (Supplementary Figure S5) as well as abiotic controls (Supplementary Figure S6) were included. Lactate and acetate were used as carbon sources and ED (Supplementary Table S2). Using the ec-MP, which allows us to perform up to 96 independent microbial electrochemical measurements, we obtained 120 data sets of chronoamperometric measurements of which 60 were included in the further microbial electrochemical analysis. The consumption of ED and the microbial composition were analyzed to derive the coulombic efficiency (*CE*) (Table 1) and the relative microbial composition in each electrochemical cell.

**TABLE 1:** Maximum current density (*j*_max_) and the time when *j*_max_ is reached (*t*_max_) as well as coulombic efficiency (*CE*) (mean ± sd): (A) *S. oneidensis* with 10 mmol L^−1^ lactate (*n* = 8); (B) *G. sulfurreducens* with 10 mmol L^−1^ acetate as ED (*n* = 8), as well as co-cultivations of *S. oneidensis* and *G. sulfurreducens* with (C) 10 mmol L^−1^ acetate (*n* = 10), (D) 10 mmol L^−1^ lactate (*n* = 8), and (E) 5 mmol L^−1^ lactate + 5 mmol L^−1^ acetate as ED (*n* = 8).

Microorganism	*j*_max_ (µA cm^−2^)	*t*_max_ (h)	CE (%)
A) *S. oneidensis*	1.7 ± 0.2	97.2 ± 20.3	5.3 ± 0.8
10 mmol L^−1^ lactate			
B) *G. sulfurreducens*	137.5 ± 6.0	76.1 ± 10.7	100.3 ± 7.5
10 mmol L^−1^ acetate			
C) *G. sulfurreducens, S. oneidensis*	167.7 ± 32.0	70.8 ± 15.7	94.8 ± 15.7
10 mmol L^−1^ acetate			
D) *G. sulfurreducens, S. oneidensis*	88.6 ± 14.0	52.7 ± 0.5	81.4 ± 6.5
10 mmol L^−1^ lactate			
E) *G. sulfurreducens, S. oneidensis*	99.36 ± 19.12	59.6 ± 1.9	93.5 ± 12.1
5 mmol L^−1^ lactate, 5 mmol L^−1^ acetate			

**FIGURE 2:**
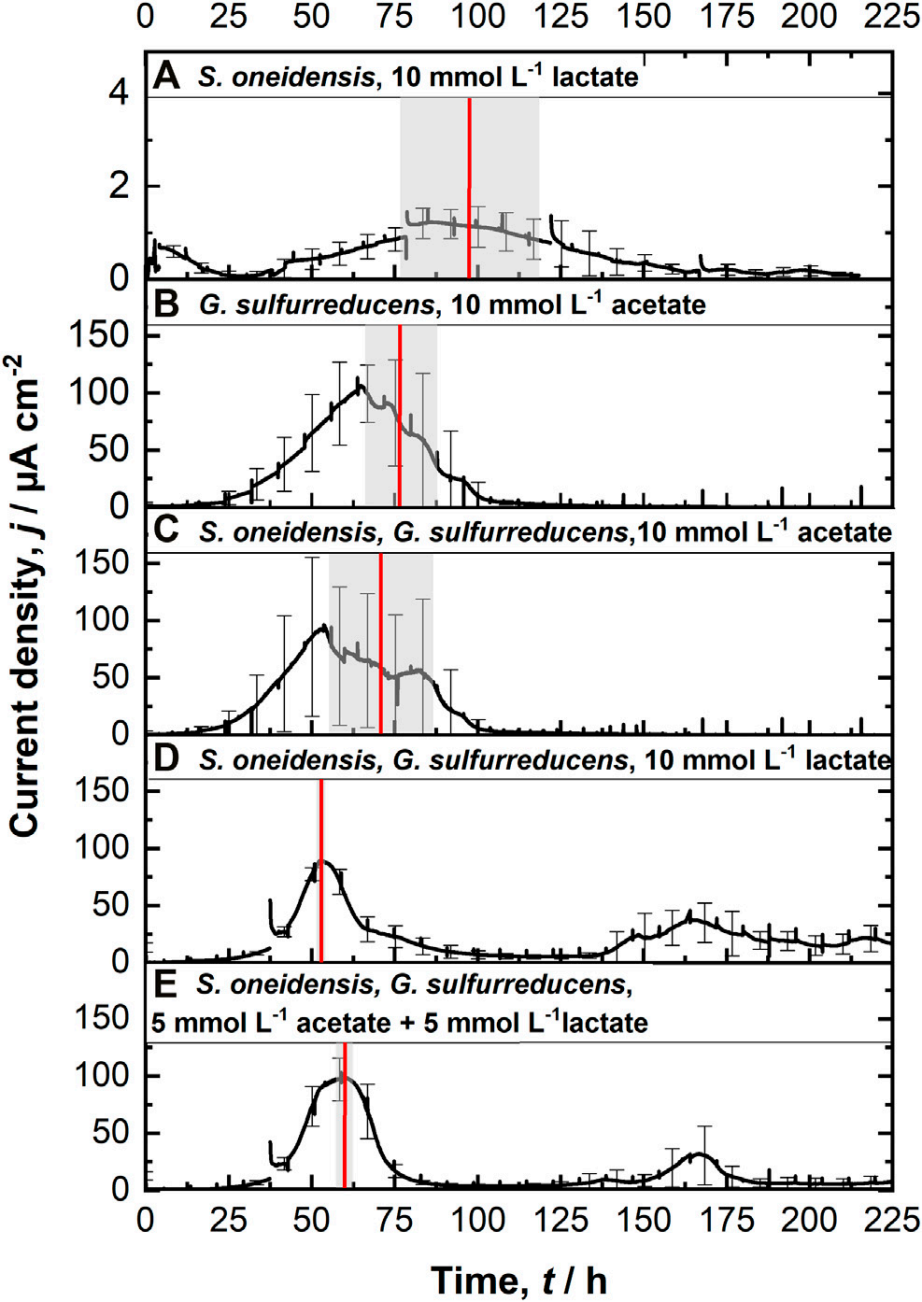
Chronoamperometric measurements (CA) at 0.4 V vs. SHE using the ec-MP with each run being independently performed in one well using a three electrode setup: **(A)** *S. oneidensis* with 10 mmol L^−1^ lactate (*n* = 8); **(B)** *G. sulfurreducens* with 10 mmol L^−1^ acetate as ED (*n* = 8), as well as co-cultivations of *S. oneidensis* and *G. sulfurreducens* with **(C)** 10 mmol L^−1^ acetate (*n* = 10), **(D)** 10 mmol L^−1^ lactate (*n* = 8), and **(E)** 5 mmol L^−1^ lactate + 5 mmol L^−1^ acetate as ED (*n* = 8). The time when maximum current density is reached *t*_max_ (red line, calculated) as well as their standard deviation (grew box) is included. For a better clarification of the high degree of reproducibility, see Supplementary Figure S7.

**FIGURE 3:**
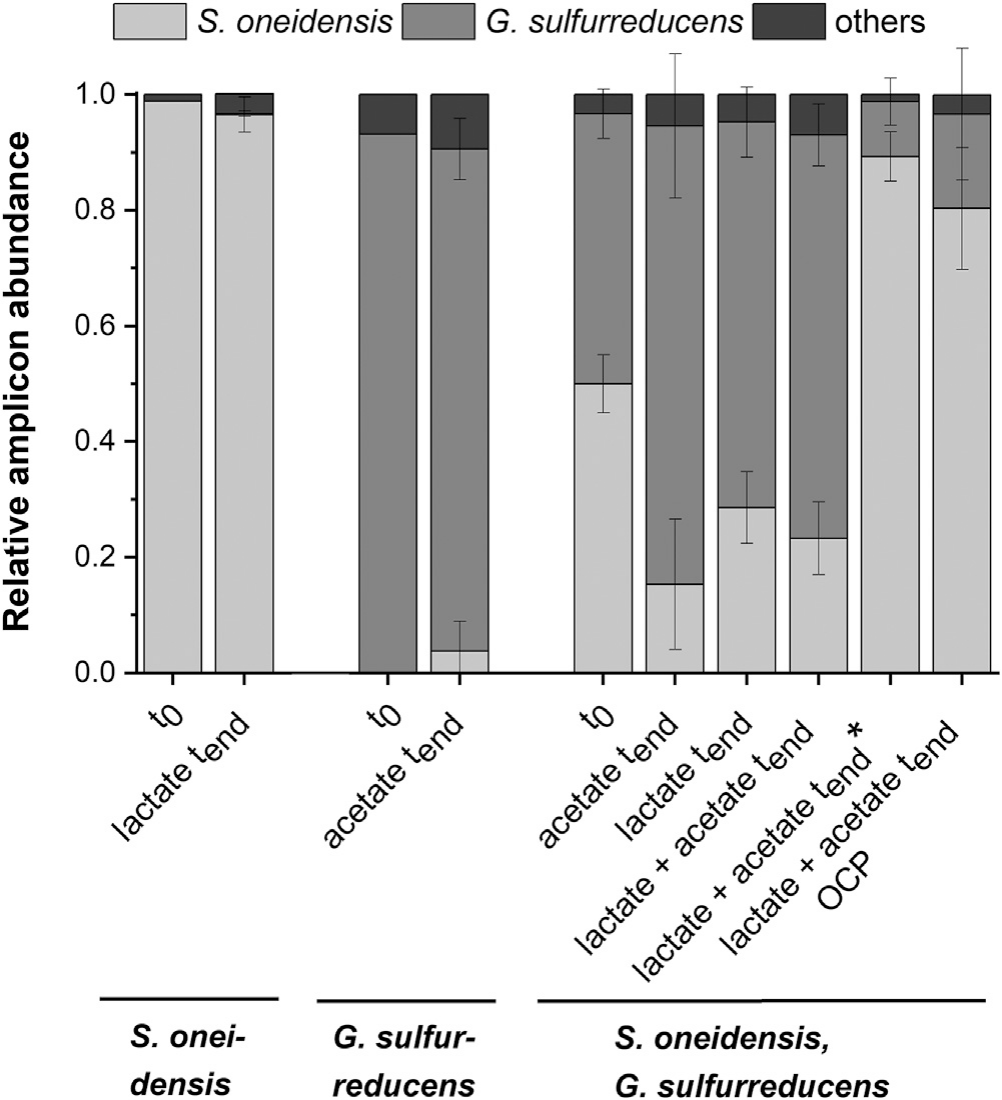
Microbial composition based on 16s rRNA TRFLP analysis (mean ± sd) for the pure cultures experiments with *S. oneidensis* (*n* = 8) and *G. sulfurreducens* (*n* = 7), the co-cultivation experiments with both strains (acetate *n* = 3; lactate *n* = 6; lactate + actete *n* = 6; lactate + actete* *n* = 2) as well as the OCP (*n* = 5) control at the start (*t*_0_; S. o. *n* = 1, G.s. *n* = 1, co-cultivation *n* = 3), and the end (*t*_end_) of the experiment (mean ± sd is not provided for other TRFs).

Figure 2A shows chronoamperometric cultivation of *S. oneidensis* being one of the most prominent EAM for MEET, which is oxidizing lactate to acetate under anaerobic conditions (Lovley 2006). The maximum current density (*j*_max_) of 1.7 ± 0.2 µA cm^−2^ at ITO after 97.2 ± 20.3 h and especially the *CE* of 5.3 ± 0.8% are consistent with studies where graphite anodes served as TEA (Rosenbaum et al., 2011; Engel et al., 2019). The CA shows a first current peak, which decreased after 24 h. We assume that possibly remaining traces of oxygen enable a complete oxidation of lactate to CO_2_ in this early phase by a few cells, gaining up to 12 electrons per molecule. When O_2_ is respired *S. oneidensis* has to switch to the anaerobic microbial electrochemical lactate oxidation, where only up to four electrons per molecule are gained. This anaerobic microbial electrochemical activity produces current densities as high as in the initial phase only later in the experiment, when higher cell numbers are reached. The stoichiometric conversion of lactate to acetate of 1:1 (HPLC data, Supplementary Table S3) confirms that *S. oneidensis* cannot use acetate as ED during anaerobic electrochemical cultivation (TerAvest et al., 2014). This result is confirmed by the microbial composition showing a high dominance of *S. oneidensis* with 96.6 ± 3.2% (Figure 3: *S. oneidensis* lactate *t*_end_).

In contrast to *S. oneidensis, G. sulfurreducens* is able to oxidize acetate during anaerobic microbial electrochemical cultivation (Lovley 2006) while forming multilayer biofilms at the anode and performing DEET. A *j*_max_ of 137.5 ± 6.0 µA cm^−2^ after 76.1 ± 10.7 h and *CE* of 100.3 ± 7.5% were reached (Figure 2B). Unfortunately, a cross-contamination of some wells (*n* = 3) serving as independent electrochemical cells occurred. *S. oneidensis* was detected in the cultivation of *G. sulfurreducens* using the ec-MP with an abundance of 3.8 ± 5.1% while the abundance of *G. sulfurreducens* was 86.8 ± 5.3% (Figure 3). We speculate that the possible initial traces of oxygen were a trigger for the establishment of *S. oneidensis*.

The co-cultivation of *S. oneidensis* and *G. sulfurreducens* was performed in the ec-MP using the equal conditions as for the pure cultures. In the co-culture experiments (Figures 2C–E) maximum current density *j*_max_ was reached after 70.8 ± 15.7 h for acetate, 52.7 ± 0.5 h for lactate, and 59.6 ± 1.9 h for lactate and acetate as ED. Thus, in co-culture, the maximum current production is reached around 23 h when lactate or 17 h earlier when lactate and acetate were used as ED compared to pure *G. sulfurreducens* with only acetate as ED (76.1 ± 10.7 h). In contrast, the co-cultivation with acetate as sole ED did not lead to a faster current production. Also the *j*_max_ itself of 167.7 ± 32.0 µA cm^−2^ for co-cultivation of *S. oneidensis* and *G. sulfurreducens* was similar to that of pure *G. sulfurreducens* (137.5 ± 6.0 µA cm^−2^). However, when using lactate as ED a clearly higher *j*_max_ of 88.6 ± 14.0 µA cm^−2^ in comparison to pure culture experiments of *S. oneidensis* (*j*_max_ of 1.7 ± 0.2 µA cm^−2^) were achieved. An increased maximum current density as reported by Engel et al. (Engel et al., 2019) for co-cultivation with lactate and acetate as ED in comparison with *G. sulfurreducens* pure culture with acetate could not be measured. The *j*_max_ of 99.4 ± 19.1 µA cm^−2^ for co-cultivation with lactate and acetate as ED is clearly reduced when compared to *G. sulfurreducens*. The mentioned values of *j*_max_ correspond to the first peaks in the CA of co-cultivation. Interestingly, when lactate or acetate and lactate served as ED in the further course of the cultivation second current peaks with a lower maximum current density (48.8 ± 20.1 µA cm^−2^ for lactate and 48.9 ± 17.2 µA cm^−2^ for lactate and acetate as ED) occurred. This may indicate a biphasic metabolism. Also Engel et al. (Engel et al., 2019) and Speers and Reguera (Speers and Reguera 2012) mentioned that after certain time lactate degradation takes place while the acetate concentration increases using graphite anodes. Nevertheless, such a strong biphasic current production and thus metabolism as we observed for ITO was to the best of our knowledge not reported before. In general, the *CE*s for the different co-cultures were all in the same range, specifically 81.4 ± 6.5% for lactate, 94.2 ± 15.7% for acetate, and 93.5 ± 12.1% for lactate and acetate as ED respectively. Reaching these high values, whether it was for pure *G. sulfurreducens* (100.3 ± 7.5%) or co-culture experiments, indicates vital and metabolic active cells. However, considering that a biomass growth occurs within this one batch cycle, a *CE* of close 100% cannot be achieved even theoretically. This and also *CE* values above 100% show that H_2_ produced at the cathode, in addition to acetate and lactate, was oxidized (Caccavo et al., 1994; Lee and Rittmann 2010; Korth et al., 2020) and thus recycled at the anode.

As Figure 3 shows a shift of abundance can be observed for all co-cultivations (*G. sulfurreducens*, *S. oneidensis*: acetate *t*_end_, lactate *t*_end_, lactate + acetate *t*_end_). Generally, in comparison to the inoculum (*t*_0_) with an equal 50% share of both microorganisms the relative abundance of *G. sulfurreducens* increased to a similar extent in which the abundance of *S. oneidensis* decreased after 10 days of cultivation (*t*_end_). The strongest shift was observed using acetate as ED leading to a change in abundance at *t*_end_ of +32.5 ± 12.5% for *G. sulfurreducens* and −34.7 ± 11.2% for *S. oneidensis*. Using lactate as ED led to a change in abundance at *t*_end_ of +20.2 ± 3.2% for *G. sulfurreducens* and of −21.4 ± 6.2% for *S. oneidensis*, whereas using acetate and lactate as ED led to +23.0 ± 5.4% for *G. sulfurreducens* and to −26.7 ± 4.2% for *S. oneidensis*. Interestingly, for two out of eight replicates of co-cultivation using combined lactate and acetate as ED showed an opposite shift that was +39.3 ± 4.3% for *S. oneidensis* and −37.3 ± 4.0% for *G. sulfurreducens* (Figure 3: *S. oneidensis*, *G. sulfurreducens* lactate + acetate *t*_end_*). Only for these two replicates the additional current peaks were not detected (Supplementary Figure S8). Further, an increased acetate concentration of 7.6 ± 1.03 mmol L^−1^ (acetate concentration *t_0_* 4.8 mmol L^−1^) is showing an almost stoichiometric accumulation from lactate oxidation to acetate. The reasons for this different shift in the community and hence the microbial electrochemical performance cannot be deciphered. They may range from increased oxygen availability by leakage into the wells to stochastic microbial processes (Zhou et al., 2013; Ofiţeru et al., 2010) including the first settling at the electrode (Kees et al., 2021). This was already discussed for microbial electrochemical selection and cultivation previously (Koch et al., 2019). In this vein it is noteworthy that co-cultivation using the ec-MP at OCP, where a complete conversion of lactate to acetate took place, showed also a shift of the microbial composition towards *S. oneidensis* with an abundance increase of 30.4 ± 10.5% (−30.4 ± 11.4% for abundance of *G. sulfurreducens*).

In summary, for co-cultivation in which lactate is involved we observed a positive effect in terms of a decreased time to reach maximum current density of around 20 h and hence faster current production that came along with a shift within the microbial composition towards *G. sulfurreducens*. Increased maximum current densities during co-cultivation were only observed when lactate or lactate and acetate as ED was provided, as *S. oneidensis* allows tapping this carbon source by *G. sulfurreducens*. A study of Prokhorova et al. (Prokhorova et al., 2017) investigated microbe-microbe and microbe-electrode interaction between *S. oneidensis*, *G. sulfurreducens* as well as *G. metallireducens* with the latter playing only a minor role in the studied consortium. They reported the upregulation of several proteins of *G. sulfurreducens,* for instance, outer membrane cytochromes, porincytochrome complex components, several membrane-associated cytochromes, and the major pilus component *pilA*, that can be assumed to foster EET within the consortium. Also for *S. oneidensis* a positive effect was determined in terms of substrate oxidation and electron transfer processes by upregulation of Mtr pathway and lactate transport and oxidation proteins (Prokhorova et al., 2017). Further findings of Okamoto et al. show that *G. sulfurreducens* is able to use (self-secreted) flavins similar to that of *S. oneidensis* for enhancing EET, when only monolayer biofilm without conductive nanowires is present (Okamoto et al., 2014). We speculate that a utilization of flavins secreted by *S. oneidensis* by *G. sulfurreducens* for EET may explain the decreased time for reaching maximum current density in co-cultures. Another fact that strengthens this hypothesis is that already the presence of *S. oneidensis* in the planktonic phase has a positive effect on *G. sulfurreducens* and the formation of thick biofilms. A positive correlation between biofilm thickness and current production could be confirmed by Engel et al. (2019), but a stable incorporation of *S. oneidensis* into the biofilm seems not to exist and seems not to be important for the further development of biofilms being dominated by *G. sulfurreducens* and thus current production. Ultimately, the clearly demonstrated benefits of co-culturing seems diverse, including foodwebs as well as enabling enhanced EET and even DIET between *S. oneidensis* and *G. sulfurreducens* cannot be ruled out, so far.

## Conclusion

We demonstrated that a high degree of parallelization of electrochemical cultivations using the ec-MP can be achieved. The cultivation of model organisms yield *j*_max_ and *CE* that are well in line with literature and the parallelization allows further insights, for instance here into a biphasic metabolism when using lactate as ED for a co-culture of *S. oneidensis* and *G. sulfurreducens*. The ec-MP presented here will allow a true parallelization of the microbial electrochemical screening and microbial electrochemically driven selection. This will open the door to perform microbial resource mining in habitats that are already well known for harbouring EAM like wastewater and soil (Koch and Harnisch 2016a; Logan et al., 2019) but also recently discovered ones like the oral (Naradasu et al., 2020) or gut (Tahernia et al., 2020b; Rago et al., 2021) microbiome and especially to explore new habitats as resources. Further, we foresee that already exploited EAM, for instance, for microbial electrosynthesis of chemical building blocks (Mayr et al., 2019; Wu et al., 2019), can be further improved using concepts and tools that are well established (for non-electrochemical means) like site directed mutagenesis, CRISPR-CAS, and techniques beyond in high-throughput (Alves et al., 2017; Fan et al., 2020). Even longer and more complex measurements will become a possibility by integrating microfluidics (Yoon et al., 2018; Yates et al., 2021; Li et al., 2011), e.g., to replenish culture media or to enable complex co-cultivation experiments.

## Data Availability

The original contributions presented in the study are included in the article/Supplementary Material, further inquiries can be directed to the corresponding author.
