# Supplementary figures and images for: Electrochemical Microwell Plate to Study Electroactive Microorganisms in Parallel and Real-Time
Source: Front Bioeng Biotechnol. 2022 Feb 15;9:821734. doi: 10.3389/fbioe.2021.821734 (PMC8887713; doi:10.3389/fbioe.2021.821734)

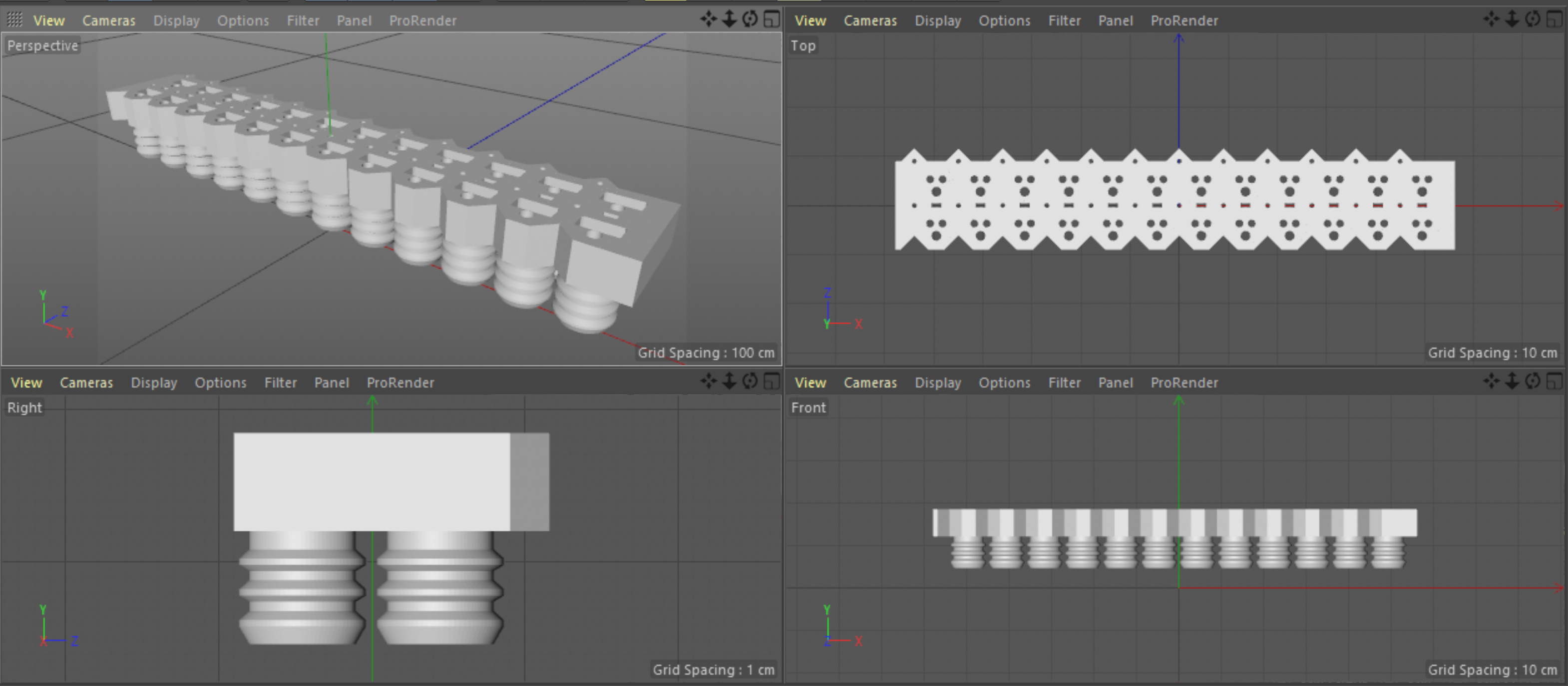

Supplement: Supplementary file 1 [file DataSheet1.ZIP › 24Well module_backbone.PNG]

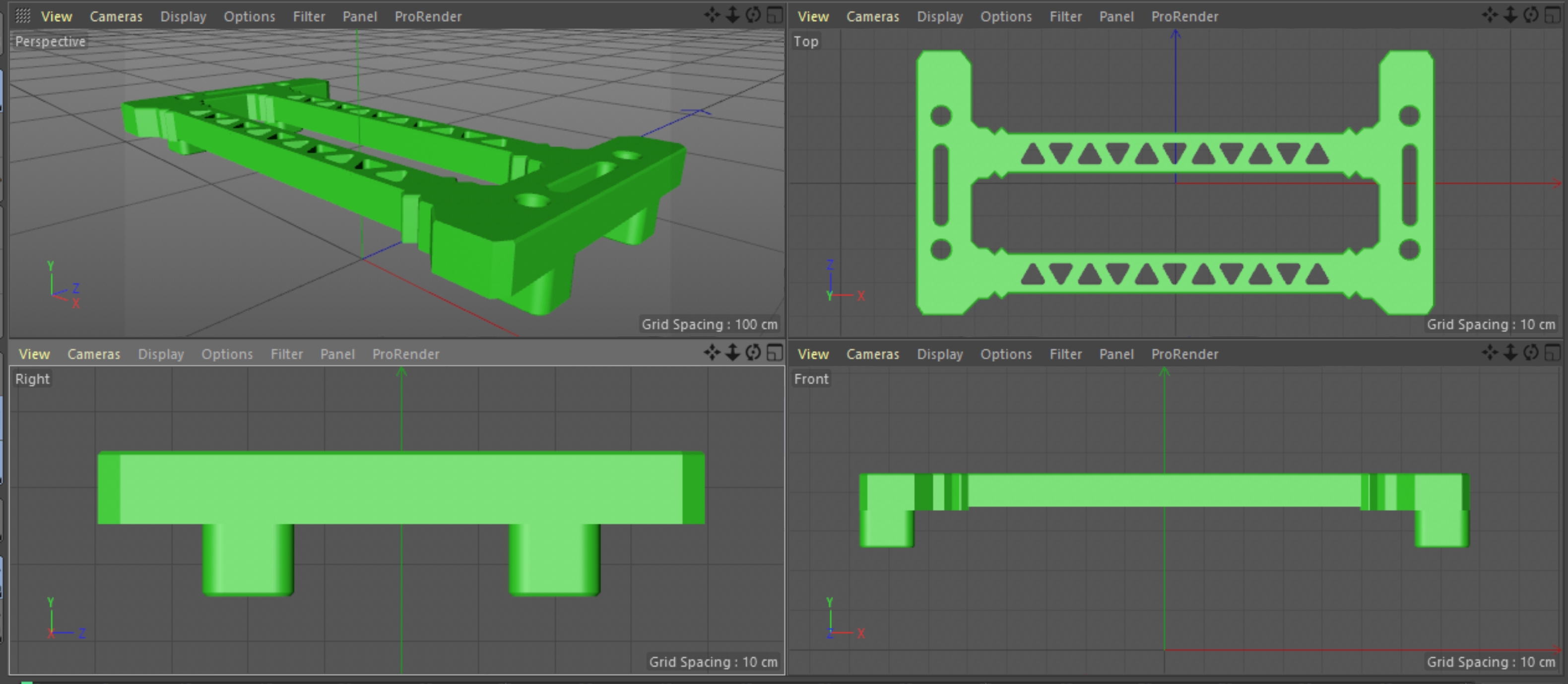

Supplement: Supplementary file 1 [file DataSheet1.ZIP › Mechanical Load.PNG]
