# Supplementary material for: Electrochemical Microwell Plate to Study Electroactive Microorganisms in Parallel and Real-Time
Source: Front Bioeng Biotechnol. 2022 Feb 15;9:821734. doi: 10.3389/fbioe.2021.821734 (PMC8887713; doi:10.3389/fbioe.2021.821734)
Supplement: Supplementary file 2 [file DataSheet2.docx]

Supplementary Material

**Electrochemical microwell plate to study electroactive microorganisms in parallel and real-time**

Anne Kuchenbuch^1, †^ , Ronny Frank^2, †^, José Vazquez Ramos^2^, Heinz-Georg Jahnke^2^, Falk Harnisch^1*^

^1^ UFZ – Helmholtz-Centre for Environmental Research GmbH, Department of Environmental Microbiology, Permoserstraße, 15, 04318 Leipzig, Germany

^2^ Centre for Biotechnology and Biomedicine, Molecular biological-biochemical Processing Technology, Leipzig University, Deutscher Platz 5, D-04103 Leipzig, Germany

*** Correspondence:**Falk Harnisch
[falk.harnisch@ufz.de](mailto:falk.harnisch@ufz.de)

^†^These authors have contributed equally to this work


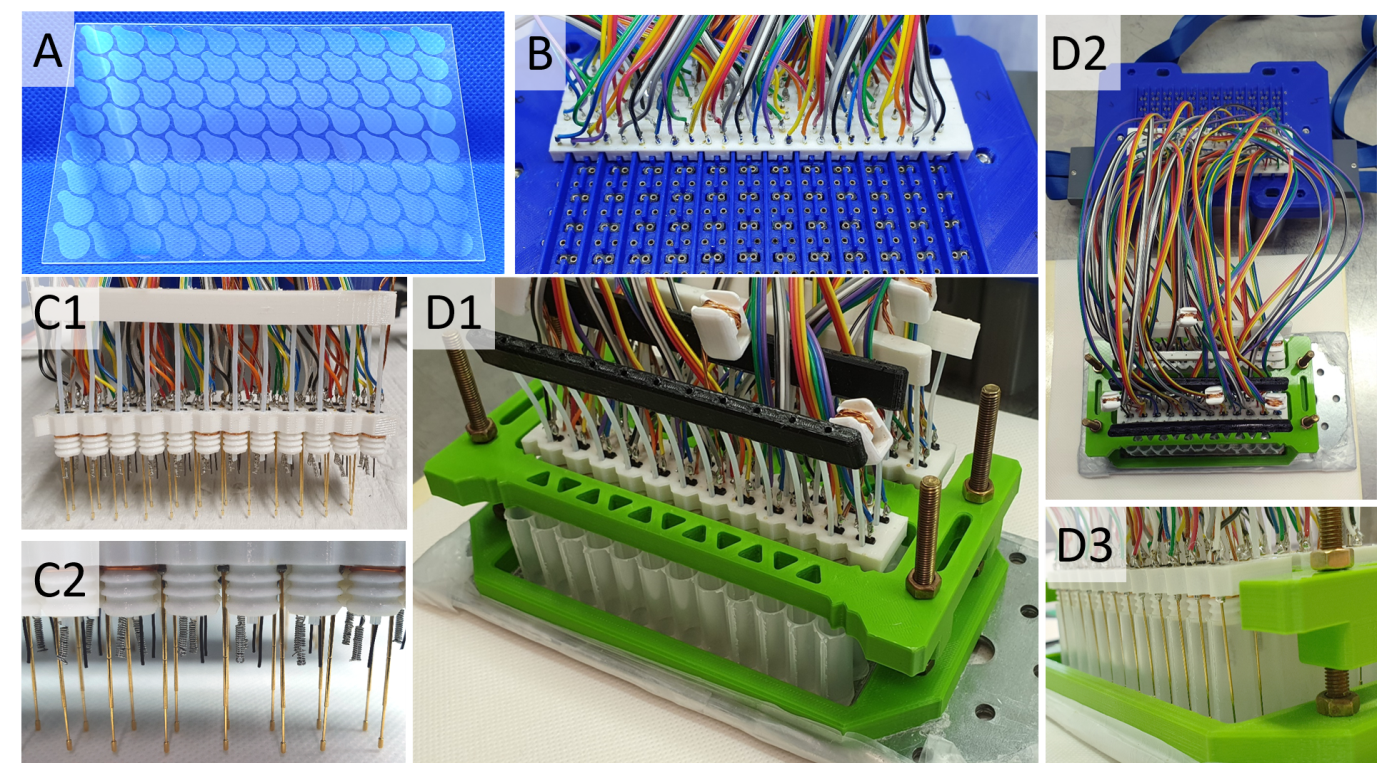


**Supplementary Figure 1.** **96-electrochemical microwell plate**. A) Indium tin oxide working electrode array on borosilicate glass substrate. B) Potentiostat mapper showing two connected 24-well modules. C) 24-well module. D) Assembled Setup with two of up to four possible 24-well modules.

**Supplementary Table 1: Medium supplements** – DSM 141 vitamin (A) and trace element (B) solution for cultivation of *G. sulfurreducens* and M4 trace element sulfate (C) and chloride (D) solution for cultivation of *S. oneidensis*

| **Concentration** | **Substance** | **Concentration** | **Substance** |
| --- | --- | --- | --- |
| 1. Vitamin solution DSM 141 | | 1. Trace element solution DSM 141 | |
| 2 mg L^-1^ | biotin | 1.50 g L^-1^ | nitrilotriacetic acid |
| 2 mg L^-1^ | folic acid | 3.00 g L^-1^ | MgSO_4_ x 7 H_2_O |
| 10 mg L^-1^ | pyridoxine- HCl | 0.50 g L^-1^ | MnSO_4_ x H_2_O |
| 5 mg L^-1^ | riboflavin | 1.00 g L^-1^ | NaCl |
| 5 mg L^-1^ | Ca-pantothenate | 0.10 g L^-1^ | FeSO_4_ x 7 H_2_O |
| 5 mg L^-1^ | p-amino benzoic acid | 0.18 g L^-1^ | CoSO_4_ x 7 H_2_O |
| 5 mg L^-1^ | lipoic acid | 0.10 g L^-1^ | CaCl_2_x 2 H_2_O |
| 5 mg L^-1^ | thiamine- HCl | 0.18 g L^-1^ | ZnSO_4_ x 7 H_2_O |
| 5 mg L^-1^ | nicotinic acid | 0.01 g L^-1^ | CuSO_4_x 5 H_2_O |
| 0.1 mg L^-1^ | cyanocobalamin | 0.02 g L^-1^ | KAl(SO_4_)_2_ x 12 H_2_O |
|  |  | 0.01 g L^-1^ | H_3_BO_3_ |
|  |  | 0.01 g L^-1^ | Na_2_MoO_4_ x 2 H_2_O |
|  |  | 0.03 g L^-1^ | NiCl_2_ x 6 H_2_O |
|  |  | 0.30 mg L^-1v^ | Na_2_SeO_3_x 5 H_2_O |
|  |  | 0.40 mg L^-1^ | Na_2_WO_4_x 2 H_2_O |
| 1. M4 trace element sulfate solution | | 1. M4 trace element chloride solution | |
| 0.5 mg L^-1^ | biotin | 2.5 g L^-1^ | nitrilotriacetic acid |
| 35.2 mg L^-1^ | folic acid | 1.5 g L^-1^ | MgSO_4_ x 7 H_2_O |
| 2.2 mg L^-1^ | pyridoxine- HCl | 119 mg L^-1^ | MnSO_4_ x H_2_O |
| 2.9 mg L^-1^ | riboflavin | 107 mg L^-1^ | NaCl |
| 9.4 mg L^-1^ | Ca-pantothenate | 0.119 mg L^-1^ | FeSO_4_ x 7 H_2_O |
| 3.9 mg L^-1^ | p-amino benzoic acid |  |  |

**Supplementary Table 2: Electrochemical cultivation** of pure culture for *S. oneidensis* (A); *G. sulfurreducens* (B); co-cultivation experiments of *G. sulfurreducens* and *S. oneidens*is (C-E) as well as OCP (F) and abiotic controls (G) under indication of applied potential, Carbon source/ ED and number of independent replicates.

| Microorganism | Applied potential  [V vs. SHE] | Carbon source/ ED | Number of replicates |
| --- | --- | --- | --- |
|  |  |  |  |
| A) *S. oneidensis* | 0.4 | 10 mmol L^-1^ lactate | 8 |
| B) *G. sulfurreducens* | 0.4 | 10 mmol L^-1^ acetate | 8 |
| C) *G. sulfurreducens, S. oneidensis* | 0.4 | 10 mmol L^-1^ acetate | 12 |
| D) *G. sulfurreducens, S. oneidensis* | 0.4 | 10 mmol L^-1^ lactate | 8 |
| E) *G. sulfurreducens, S. oneidensis* | 0.4 | 5 mmol L^-1^ lactate, 5 mmol L^-1^ acetate | 8 |
| F) *G. sulfurreducens, S. oneidensis* | OCP* | 5 mmol L^-1^ lactate, 5 mmol L^-1^ acetate | 8 |
| G) Abiotic control | 0.4 | 5 mmol L^-1^ lactate, 5 mmol L^-1^ acetate | 8 |

*OCP is open circuit potential


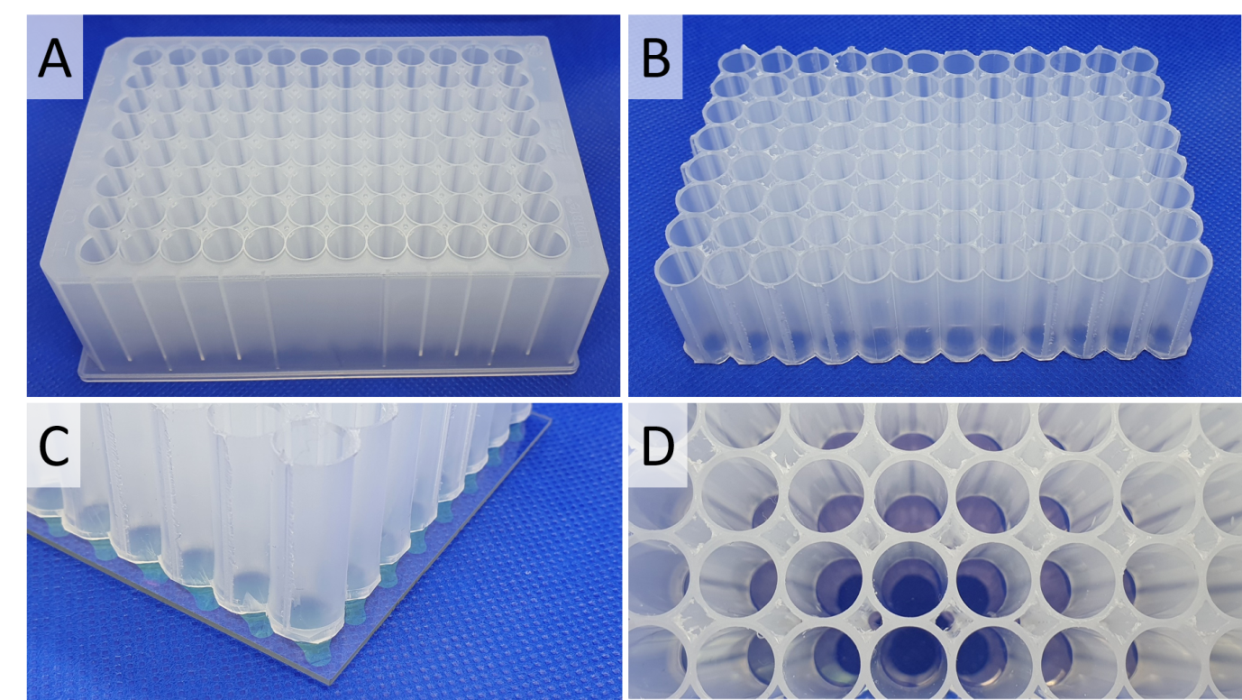


**Supplementary Figure 2. 96-deepwell module.** A) 2 mL deepwell Riplate^TM^. B) Modified deepwell plate without bottom and with open interspaces. C+D) deepwell plate bonded to a working electrode array.


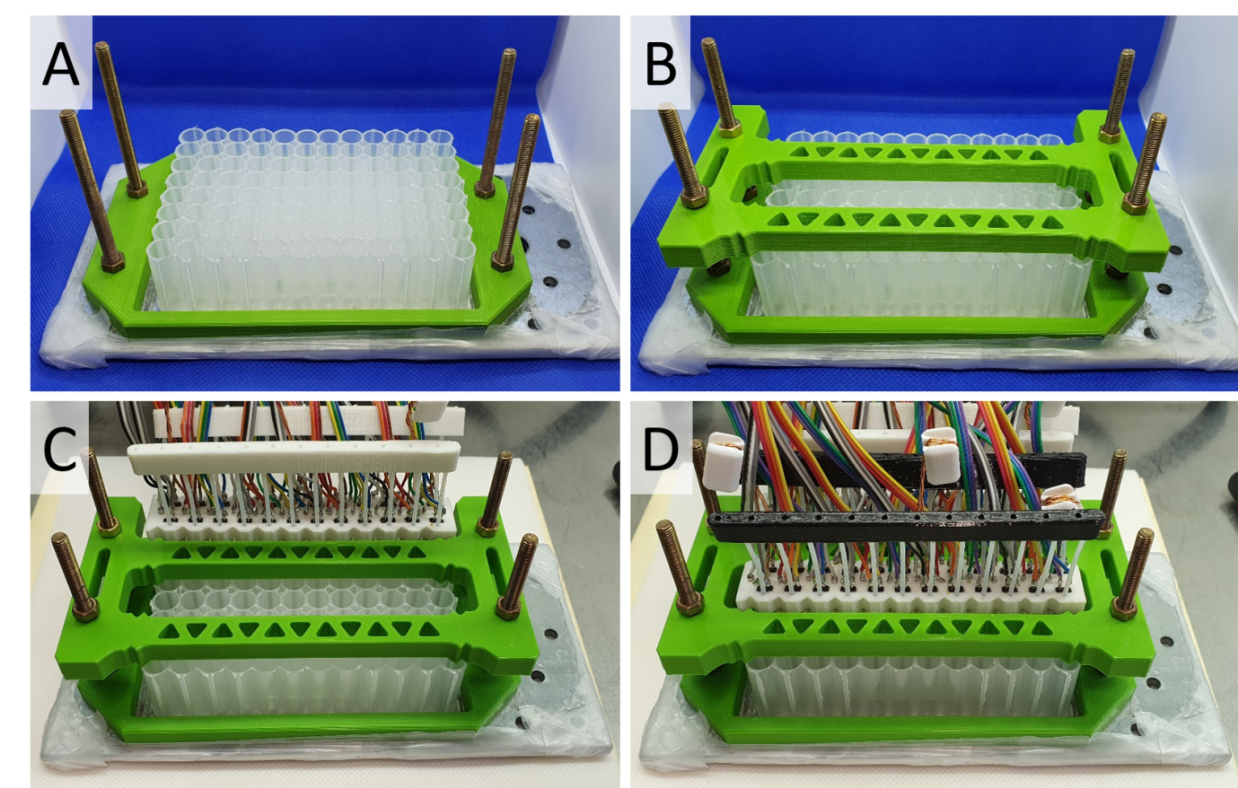


**Supplementary Figure 3. Assembly of the electrochemical microwell plate** (ec-MP). A) deepwell module is placed in the assembly device and fixed to avoid any movement (B). C) Setup with a single 24-well module, and with two 24-well modules (D).

**Supplementary Figure 4:** **Microbial composition based on 16s rRNA TRFLP analysis** for the pure cultures experiments with *S. oneidensis* and *G. sulfurreducens* at the start (*t*_0_) of the experiment (n=1).

**Supplementary Figure 5.** Chronoamperometric measurement at open circuit potential (OCP)

**Supplementary Figure 6. Chronoamperometric measurement of the abiotic control** at 0.4 V vs. SHE with 5 mmol L^-1^ lactate and 5 mmol L^-1^ acetate as ED


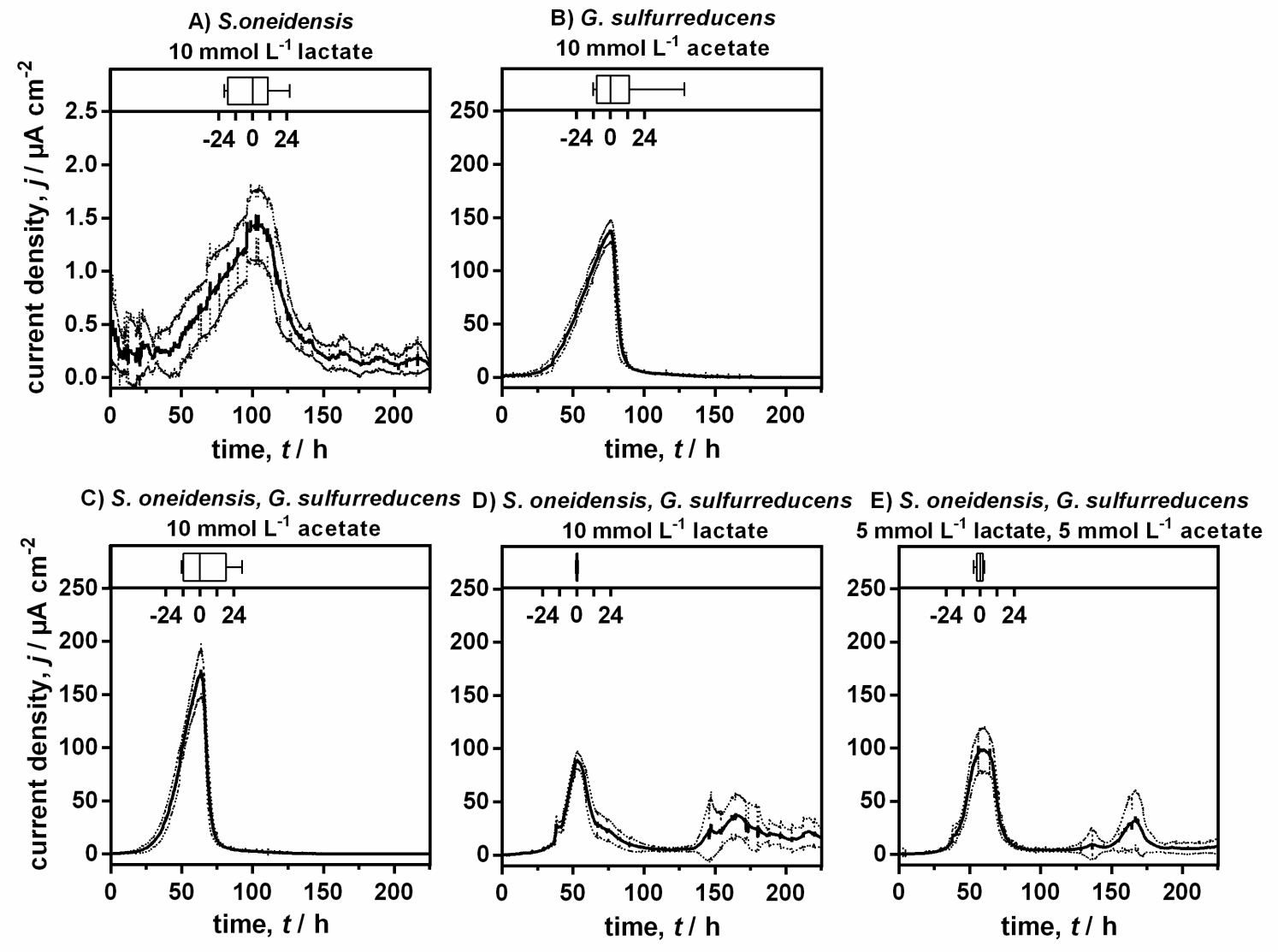


**Supplementary Figure 7. Aligned chronoamperometric measurements (CA), equal to Figure 2, at 0.4 V vs. SHE using the ec-MP with each run being independently performed in one well using a three electrode setup.** A) *S. oneidensis* with 10 mmol L^-1^ lactate (n=8) B) *G. sulfurreducens* with 10 mmol L^-1^ acetate as ED (n=8), as well as co-cultivations of *S. oneidensis* and *G. sulfurreducens* with C) 10 mmol L^-1^ acetate (n=10), D) 10 mmol L^-1^ lactate (n=8) and E) 5 mmol L^-1^ lactate + 5 mmol L^-1^ acetate as ED (n=8). Alignment procedure: From each run the maximum current density was determined to calculate the median of the maximum current densities of all replicates. Chronoamperometric data of each run were then shifted in the time axis to the median of the maximum current densities. From the shift magnitude, a boxplot was generated reflecting the distribution of the maximum current densities that might originate from different lag phases. (aligned CA data, mean±SD, SD is shown as dotted lines)

**Supplementary Table 3. HPLC data** - concentrations of the ED in mmol L^-1^ (mean±sd) of the chronoamperiometric cultivation of *S. oneidensis* and *G. sulfurreducens* as pure culture with 10 mmol L^-1^ lactate and 10 mmol L^-1^ acetate respectively, co-cultivation of both strains with 10 mmol L^-1^ lactate, 10 mmol L^-1^ acetate as well as 5 mmol L^-1^ lactate and 5 mmol L^-1^ acetate as ED and the OCP as well as abiotic control at time point t_0_ and t_end_. *acetate concentration t_end_ of two out of eight replicates of co-cultivation of both strains with 5 mmol L^-1^ lactate and 5 mmol L^-1^ acetate as ED (see **Figure S4B**).

| Microorganism | Carbon Souce/ ED | substance | Concentration mmol L^-1^  t_0_ | Concentration mmol L^-1^  t_end_ |
| --- | --- | --- | --- | --- |
| A) *S. oneidensis* | 10 mmol L^-1^ lactate | lactate  acetate | **9.6±0.0**  **0** | **0**  **9.4±3.4** |
| B) *G. sulfurreducens* | 10 mmol L^-1^ acetate | lactate  acetate | **0**  **9.8±0.4** | **0**  **0** |
| C) *G. sulfurreducens,*  *S. oneidensis* | 10 mmol L^-1^ acetate | lactate  acetate | **0**  **10.0±0.5** | **0**  **0.1±0.3** |
| D) *G. sulfurreducens,*  *S. oneidensis* | 10 mmol L^-1^ lactate | lactate  acetate | **9.6±0.0**  **0** | **0**  **1.0±0.9** |
| E) *G. sulfurreducens,*  *S. oneidensis* | 5 mmol L^-1^ lactate,  5 mmol L^-1^ acetate | lactate  acetate | **4.7±0.0**  **4.8±0.0** | **0**  **0.5±0.2**  **7.6±1.0*** |
| F) OCP | 5 mmol L^-1^ lactate,  5 mmol L^-1^ acetate | lactate  acetate | **4.9±0.1**  **5.0±0.3** | **0.1±0.1**  **8.2±1.2** |
| G) Abiotic control | 5 mmol L^-1^ lactate,  5 mmol L^-1^ acetate | lactate  acetate | **4.9±0.1**  **5.0±0.3** | **5.2±0.1**  **5.4±0.1** |

**A**

**B**

**Supplementary Figure 8. Chronoamperometric measurement** at 0.4 V vs. SHE of co-cultivation of *S. oneidensis* and *G. sulfurreducens* with 5 mmol L^-1^ lactate and 5 mmol L^-1^ acetate as ED of A) all eight replicates and B) two of eight replicates behaving differently
